# Temporal Convolutional Networks for the Advance Prediction of ENSO

**DOI:** 10.1038/s41598-020-65070-5

**Published:** 2020-05-15

**Authors:** Jining Yan, Lin Mu, Lizhe Wang, Rajiv Ranjan, Albert Y. Zomaya

**Affiliations:** 1 School of Computer Science, China University of Geosciences, Wuhan, 430074 China; 2 College of Life Sciences and Oceanography, Shenzhen University, Shenzhen, 518060 China; 3 Shenzhen Research Institute, China University of Geosciences, Shenzhen, 518057 China; 4 Computer Science and the Internet of Things, Newcastle University, Newcastle on Tyne, United Kingdom; 5 School of Computer Science, University of Sydney, NSW, Australia

**Keywords:** Environmental sciences, Ocean sciences

## Abstract

El Niño-Southern Oscillation (ENSO), which is one of the main drivers of Earth’s inter-annual climate variability, often causes a wide range of climate anomalies, and the advance prediction of ENSO is always an important and challenging scientific issue. Since a unified and complete ENSO theory has yet to be established, people often use related indicators, such as the Niño 3.4 index and southern oscillation index (SOI), to predict the development trends of ENSO through appropriate numerical simulation models. However, because the ENSO phenomenon is a highly complex and dynamic model and the Niño 3.4 index and SOI mix many low- and high-frequency components, the prediction accuracy of current popular numerical prediction methods is not high. Therefore, this paper proposed the ensemble empirical mode decomposition-temporal convolutional network (EEMD-TCN) hybrid approach, which decomposes the highly variable Niño 3.4 index and SOI into relatively flat subcomponents and then uses the TCN model to predict each subcomponent in advance, finally combining the sub-prediction results to obtain the final ENSO prediction results. Niño 3.4 index and SOI reanalysis data from 1871 to 1973 were used for model training, and the data for 1984–2019 were predicted 1 month, 3 months, 6 months, and 12 months in advance. The results show that the accuracy of the 1-month-lead Niño 3.4 index prediction was the highest, the 12-month-lead SOI prediction was the slowest, and the correlation coefficient between the worst SOI prediction result and the actual value reached 0.6406. Furthermore, the overall prediction accuracy on the Niño 3.4 index was better than that on the SOI, which may have occurred because the SOI contains too many high-frequency components, making prediction difficult. The results of comparative experiments with the TCN, LSTM, and EEMD-LSTM methods showed that the EEMD-TCN provides the best overall prediction of both the Niño 3.4 index and SOI in the 1-, 3-, 6-, and 12-month-lead predictions among all the methods considered. This result means that the TCN approach performs well in the advance prediction of ENSO and will be of great guiding significance in studying it.

## Introduction

El Niño-Southern Oscillation (ENSO) is a sea surface temperature and air pressure shock that occurs in the equatorial Pacific Ocean^1^. It is a sea-air interaction phenomenon at low latitudes, which is manifested by the El Niño-La Niña transition in the ocean and the “southern oscillation” (SO) in the atmosphere. El Niño refers to the warming phenomenon that occurs in the tropical Pacific every 2–7 years, while the cooling phenomenon is called La Niña^2^. El Niño and La Niña are closely related to SO, which is the inverse-change phenomenon of the pressure field in the tropical east Pacific and tropical east Indian Ocean. The ENSO is one of the main drivers of Earth’s inter-annual climate variability. It often causes a wide range of climate anomalies, triggering a variety of meteorological disasters and causing huge economic property damage in affected areas^3^.

Scientists from all over the world pay close attention to the ENSO event and provide various explanations for its causes, including self-sustained oscillatory theory, equatorial high-frequency zonal wind forcing theory, etc. However, a unified, complete ENSO theory has yet to be established, with the prediction and understanding of ENSO progression still presenting a challenge to scientists^4^. Therefore, people often use related indicators to predict the development trends of ENSO through appropriate numerical simulation models.

In general, the commonly used ENSO indexes include the Niño $3.4$ index^5^, oceanic niño index (ONI)^6^, southern oscillation index (SOI)^7^, sea-surface temperature (SST) index^8^, wind index^9^, and outgoing longwave radiation (OLR) indexes^10^. Each index is a comprehensive reflection of complex climate change factors, and people try to reveal the underlying complex climate change characteristics by studying the change law of the index. For example, the Niño $3.4$ index and ONI track the SST anomalies in the east-central tropical Pacific between ${5}^{\circ }$S $-{5}^{\circ }$N and ${170}^{\circ }$W$-{120}^{\circ }$W, namely, Niño $3.4$ region, wind index measures the movement of air flow in the upper and lower branches of the Pacific Walker circulation, and the OLR index indicates the extent of convection across the tropical Pacific. However, ENSO is a very complex phenomenon, it is difficult to use a unified index to characterize the ENSO phenomenon in different parts of the world. In general, the Niño $3.4$ index and the ONI are the most commonly used indexes to define El Niño and La Niña events in the sea, and the SOI is the oldest indicator of the ENSO state in the atmosphere^11^, which constitute the two important and highly-related components of ENSO. In addition, because the ONI is the three-month running mean of SST anomalies in the Niño $3.4$ region^12^, that is, the ONI is the three-month-moving-average of the Niño $3.4$ index. Hence, in this paper, we choose the Niño $3.4$ index as an indicator of ENSO events in the ocean and the SOI as a measure of ENSO events in the atmosphere.

For the numerical simulation models used for ENSO prediction, three general approaches exist: statistics-based methods, ML-based methods, and a hybrid approach, i.e., the statistics-ML method.

- The statistical ENSO prediction methods leverage the collation, induction, and analysis of historical ENSO indexes to realize the analysis and prediction of ENSO phenomena. Typical methods include the Holt-Winters (HW) method and the autoregressive integrated moving average (ARIMA) method. The HW method is a statistical short-term method^13^ that has been used to forecast time series with seasonal patterns and repetitive forms and uses a technique called “exponential smoothing” that reduces fluctuations in the time-series data, thus providing a clearer view of their fundamentals^14^. In 2014, Mike and Ray used the HW method to make 1-step-ahead and 12-step-ahead forecasts of the Niño region 3 SST index from January 1933 to December 2012. The final predicted out-of-sample root mean square errors of the HW model were 0.303 and 1.309, respectively. Hence, they introduced an improved HW model called the dynamic seasonality model (DSM) to alleviate the shortcomings of the HW method unsuitable for periodically stationary time series^15^. The ARIMA aims to describe the autocorrelations in time-series data. In 2011, Matthieu *et al*. developed a time-series analysis method using the ARIMA to investigate temporal correlations between the monthly Plasmodium falciparum case numbers and ENSO as measured by the SOI at the Cayenne General Hospital between 1996 and 2009. The results showed a positive influence of El Niño at a lag of three months on Plasmodium falciparum cases (p < 0.001), and the incorporation of SOI data in the ARIMA model reduced the Akaike information criterion (AIC)^16^ by 4%^7^. However, the ARIMA cannot return an estimate of the seasonal component^17^. To undertake further analysis based on the seasonal component, ARIMA models may not be the best choice.
- The ML-based ENSO prediction methods are realized by learning and mining the historical ENSO index features and establishing a prediction model for ENSO prediction. Commonly used methods include support vector regression (SVR)^18,19^, artificial neural networks (ANNs)^20,21^, long short-term memory (LSTM)^22,23^, and so on^24^. For example, in 2009, Silestre and William used a Bayesian neural network (BNN) and SVR, two non-linear regression methods, to forecast the tropical Pacific SST anomalies at lead times ranging from 3 to 15 months using the sea-level pressure (SLP) and SST as predictors. The results showed that the BNN model gave better overall forecasts than did SVR. In 2011, Ravi *et al*. selected the Niño 1 + 2, Niño 3, Niño 3.4, and Niño 4 indexes as predictors of the Indian summer monsoon rainfall index (ISMRI) using an ANN model for prediction. The results suggested that the ANN model had better predictive skills than all the linear regression models investigated, implying that the relationship between the Niño indexes and the ISMRI is essentially non-linear in nature^25^. In 2017, Zhang *et al*.^26^ adopted LSTM to predict the SST of the Bohai Sea. The comparative experimental results with SVR showed that the LSTM network achieved better prediction performance. In 2018, Clifford *et al*. took an approach based on using various complex network metrics extracted from climate networks with an LSTM neural network to forecast ENSO phenomena. The preliminary experiments showed that training an LSTM model on a network-metrics time-series data set provides great potential for forecasting ENSO phenomena multiple longer steps in advance^27^. However, the following problems still exist: (i) Although SVR does not involve non-linear optimization and cannot generate multiple minimums, as well as having good robustness to outliers^28^, the overall prediction effect of SVR is generally worse than ANNs^29^, and (ii) ANNs and LSTM both have great potential to forecast ENSO phenomena multiple steps in advance^27^ but become complex and extremely time-consuming as the number of network layers increases. Recent results, however, indicate that LSTM cannot handle the ultra-long-term dependency problem well.
- The typical practice of the hybrid (statistics-ML-based) approach is to use statistical theory to decompose time-series data; use ML methods to filter, analyse, and predict the decomposition; and finally merge the prediction results of each decomposition part. The commonly used combination algorithms include ARIMA-ANNs and ensemble empirical mode decomposition (EEMD)-convolutional long short-term memory (ConvLSTM). For example, in 2016, Patil and Deo^30^ combined numerical estimations and the ANN technique to predict the SST. They achieved accurate SST predictions of daily, weekly, and monthly values over five time steps in the future at six different locations in the Indian Ocean. In 2018, Peter *et al*. proposed a hybrid model that combines the classical ARIMA technique with an ANN to improve El Niño predictions. The 6-month-lead prediction results of the hybrid model gave slightly better forecasts than those of the National Centers for Environmental Prediction (NCEP), and the 12-month-lead prediction had similar predictive power to that of shorter-lead-time predictions^31^. In 2019, Yuan *et al*. proposed an effective neural network model, EEMD-ConvLSTM, which was based on ConvLSTM and EEMD^32^, to predict the North Atlantic oscillation (NAO) index. The experimental results showed that EEMD-ConvLSTM not only had the highest reliability according to the evaluation metrics but could also better capture the variation trends of the NAO index data^33^. However, the prediction results of these methods often depend largely on the statistical decomposition model. If the statistical decomposition model can separate the components of the time-series data well and then select an excellent time-series prediction algorithm, it will deliver better prediction results.

However, the ENSO phenomenon is a highly complex and dynamic model involving different aspects of the ocean and the atmosphere over the tropical Pacific^34^, and the variation trends over time are non-linear. The statistical method tends to have a poor fitting effect on non-linear data sets and is not ideal for complex pattern recognition and knowledge discovery. The ML-based methods, especially those based on deep-level networks, tend to be complex and computationally time-consuming and are not very predictive of very-long-term-sequence ENSO indexes. In addition, for the long time-series Niño $3.4$ index and SOI data, they not only have the characteristics of approximately periodic interannual changes but also a large amount of high-frequency random noise due to seasonal changes, which seriously reduces the numerical simulation models’ forecasting ability. Hence, it is still difficult to predict ENSO event at lead times of more than one year^5^. Therefore, choosing a novel time-series analysis model that can accurately predict the ENSO state at lead times of more than one year will be of great significance.

The temporal convolutional network (TCN), as a variant of the convolutional neural network (CNN), employs casual convolutions and dilations; hence, it is suitable for sequential data with temporality and large receptive fields. In addition, the CNN has been reported to predict the ENSO phenomenon and achieve good results^5^. However, the inherent shortcomings of the CNN, including the fixed-size input vector and inconsistent input and output sizes, limit its application in time-series prediction. Furthermore, the TCN has a simple network structure and outperforms canonical recurrent networks, such as the recurrent neural network (RNN) and LSTM networks, in terms of the accuracy and efficiency of time-series data analysis. In addition, the ensemble empirical mode decomposition (EEMD) not only can decompose high-frequency time series into some adaptive orthogonal components, called intrinsic mode functions (IMFs), but also has the advantages of noise-assistance and overcoming the drawbacks of mode mixing in conventional empirical mode decomposition (EMD)^35^. EEMD can be used to decompose the high-frequency time-series Niño $3.4$ index and SOI data into multiple adaptive orthogonal components to improve the prediction accuracy of the model. Therefore, this paper proposes the EEMD-TCN hybrid approach, which is used to decompose the highly variable ENSO indexes (Niño $3.4$ index and SOI) into relatively flat subcomponents, and then uses the TCN model to predict each subcomponent in advance, finally combining the sub-prediction results to obtain the final ENSO prediction results.

## Results

### Data

To verify the effectiveness of our proposed EEMD-TCN-based ENSO prediction approach, we selected the Niño $3.4$ index^36^ and SOI reanalysis data from 1871 to 2019 for long time-series prediction experiments. The Niño $3.4$ index and SOI reanalysis data were both downloaded from the official website of the NOAA^37^. In addition, to fully verify the robust performance of the model for long-term ENSO index prediction while eliminating the possible influence of oceanic memory in the training period on the ENSO in the validation period, the data from 1871 to 1973 were used to train the model, and the data from 1984 to 2019 were used for testing.

### Niño 3.4 index prediction results and discussion

During the model-training process, we set the maximum number of training sessions to 7000 and compared the trend of the training loss with the training times. The result was that for any one of the IMFs, as the number of training sessions increased, the loss value gradually decreased and stabilized after 2000 training sessions. Therefore, we believed that the TCN model after 2000 training sessions was stable and could be used for Niño $3.4$ index prediction. In the model prediction process, we calculated the Pearson correlation coefficient (PCC) and the root mean square error (RMSE) between the resulting predicted and actual values^38^ to evaluate the predictive performance of the model. The PCC is a measure of the linear correlation between the predicted value and the actual value, while the RMSE tries to measure their differences. The PCC and RMSE can well measure the homogeneous and heterogeneous relationship between the predicted value and the actual value and are one of the frequently used combinations to evaluate the predictive performance of a model. The formulas for calculating the PCC and RMSE are as follows:

1 $${\rm{PCC}}=\frac{\mathop{\sum }\limits_{{\rm{i}}=1}^{m}({o}_{i}-\overline{o})\cdot ({p}_{i}-\overline{p})}{\sqrt{\mathop{\sum }\limits_{i=1}^{m}{({o}_{i}-\overline{o})}^{2}}\cdot \sqrt{\mathop{\sum }\limits_{i=1}^{m}{({p}_{i}-\overline{p})}^{2}}}$$

2 $${\rm{RMSE}}=\sqrt{\frac{\mathop{\sum }\limits_{i=1}^{m}{({o}_{i}-{p}_{i})}^{2}}{m}}$$

where *m* is the length of the time-series, *p* is the prediction results and $\overline{p}$ is its mean value, $o$ represents the actual value and $\overline{o}$ represents its mean value.

**Figure 1:**
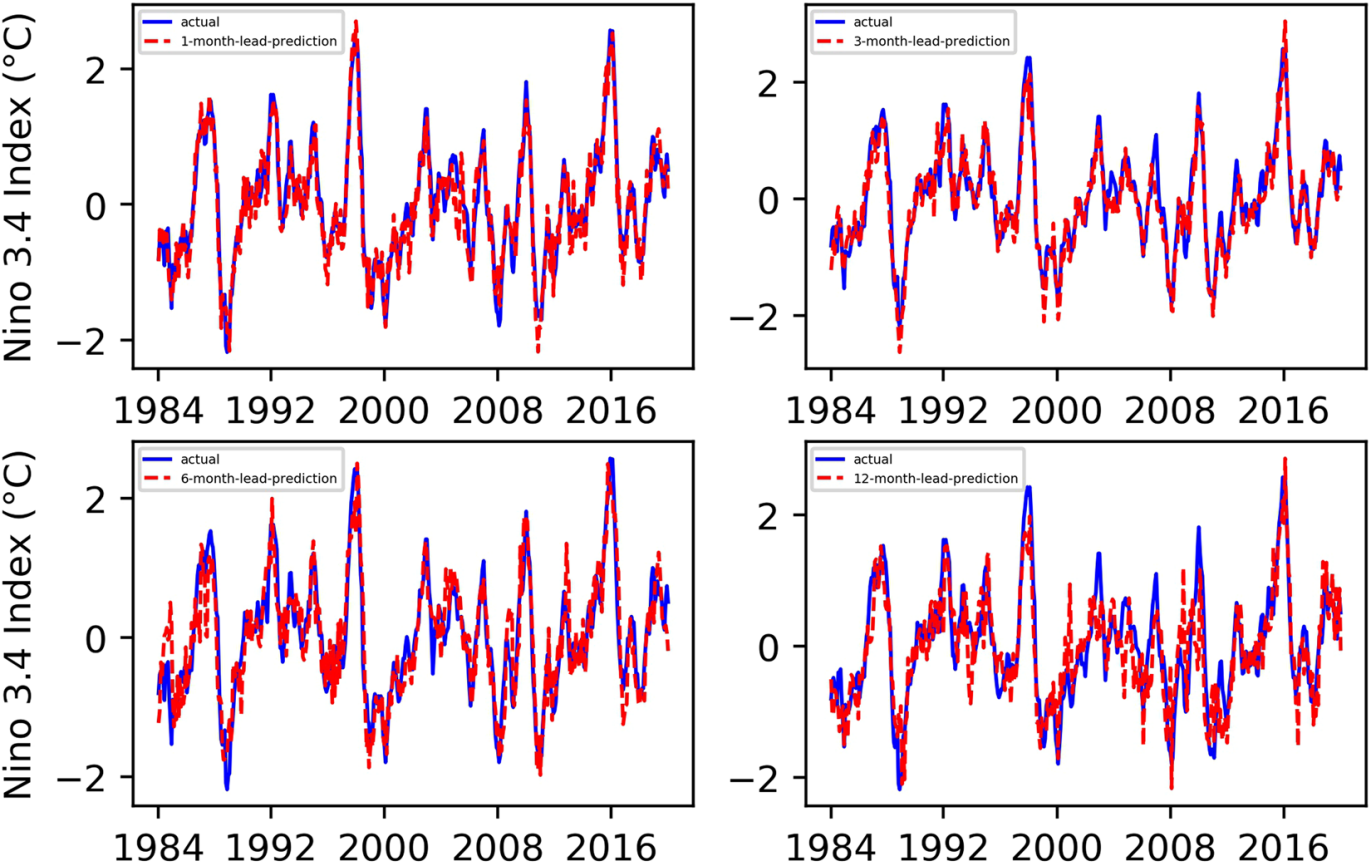
Predicted and actual values of the EEMD-TCN-based Niño $3.4$ index for different month lead times.

**Figure 2:**
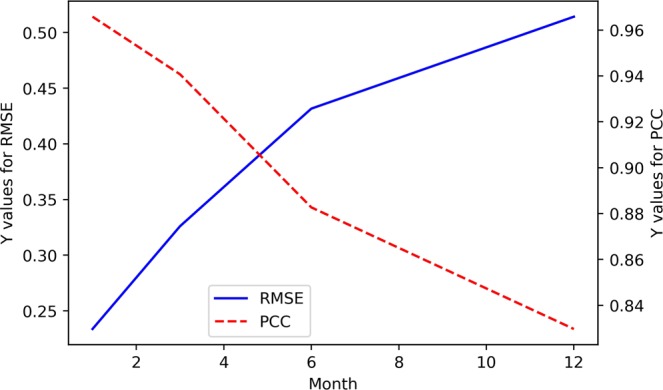
The RMSE and PCC values between the predicted and actual values in the EEMD-TCN-based Niño $3.4$ index prediction.

We conducted 1-, 3-, 6-, and 12-month-lead Niño $3.4$ index prediction experiments, and the resulting prediction results, as well as their evaluation results, are shown in Figs. 1 and 2.

From Figs. 1 and 2, (1) all the predicted Niño $3.4$ index curves almost had the same growth trend and turning points as those of the actual curve; (2) with the increase in advance prediction time, the RMSE gradually increased and the PCC gradually decreased overall, but they did not maintain strict linear variation characteristics; (3) the RMSE values of the 3- and 6-month-lead-predictions were significantly higher, while the PCC values were significantly lower, which was in line with the phenomenon of “spring forecast obstacles” in dynamic forecasting; (4) the curve obtained from the one-month-lead prediction had the highest degree of coincidence with the actual Niño $3.4$ index, the RMSE was 0.2337, and the corresponding PCC was 0.9658; (5) the curve obtained from the 12-month-lead prediction matched the actual curve the least, the RMSE was 0.5142, and the corresponding PCC was 0.8297; although the accuracy of the predicted results in the 12-month-lead case was relatively low, the same growth trend and turning point as for the actual curve could still be maintained; (6) with the increase in the advance prediction time, the forecasting deviation in some years slightly increased, such as in 1987, 1998, 2003, 2009, 2016, etc.; this may be due to the extreme El Niño and La Niña events in these years, which posed huge challenges to the predictive models; and (7) in terms of the Niño $3.4$ index advance prediction alone, the prediction accuracy of the EEMD-TCN method was similar to or slightly better than that of the previous research^5^, which reflected the effectiveness of the TCN in Niño $3.4$ index prediction.

### SOI prediction results and discussion

**Figure 3:**
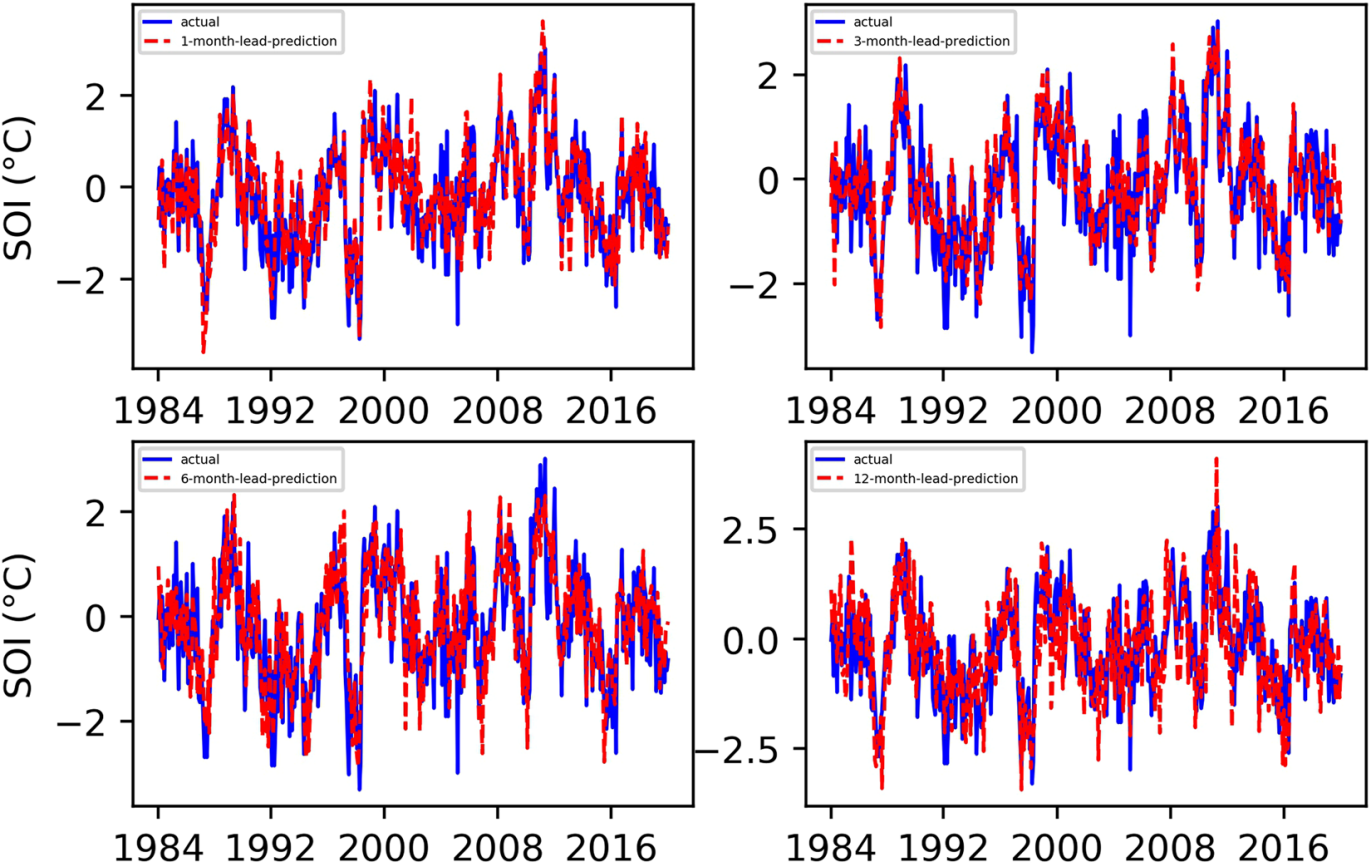
Predicted and actual values of the EEMD-TCN-based SOI for different month lead times.

**Figure 4:**
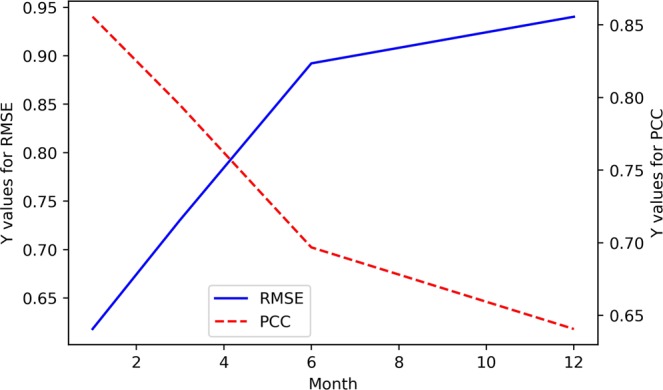
The RMSE and PCC values between the predicted and actual values in the EEMD-TCN-based SOI prediction.

Based on the Niño $3.4$ index prediction experience, we also set the maximum number of training sessions to 7000 in the SOI prediction experiment. Figures 3 and 4 show the final predicted and evaluated results.

**Figure 5:**
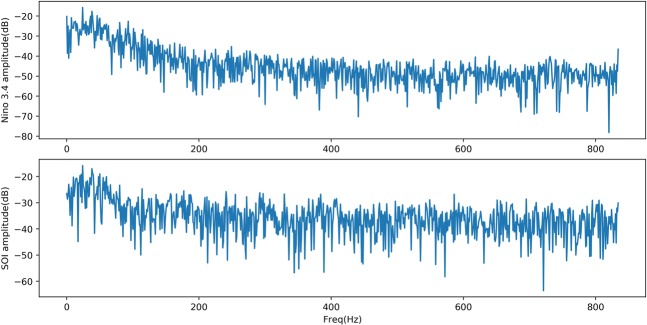
The frequency spectrum of the Niño $3.4$ index and SOI. The horizontal axis represents the frequency, and the vertical axis represents the amplitude corresponding to the frequency. The components of the SOI in the high-frequency range are significantly stronger than those of the Niño $3.4$ index.

From Figs. 3 and 4, (1) the fitting degree between the predicted and actual SOI curves of the one-month-lead prediction was the highest, and the predicted SOI curve almost had the same growth trend and turning points as those of the actual curve; (2) the RMSE of the one-month-lead prediction was 0.6180, and its corresponding PCC was 0.8556; (3) with the increase in the advance prediction time, the PCC gradually decreased, but the RMSE curve showed a tortuous trend, which may be due to the influence of the phenomenon of “spring forecast obstacles” in dynamical forecasting; (4) the accuracy of the 12-month-lead prediction was the worst, with an RMSE value of 0.9403 and a PCC value of 0.6406; and (5) as a whole, the prediction accuracy of the EEMD-TCN approach on the SOI was worse than that on the Niño $3.4$ index, which may be due to the significant difference between the two in the frequency domain (Fig. 5). The Niño $3.4$ index has significant interannual quasi-period peaks within 3 to 6 years. These peaks occur because the equatorial Kelvin waves and Ross Bay waves that determine the El Niño phenomenon in the ocean take approximately 2 years to complete adjustments in the Pacific Basin. The SOI, which is the response of the El Niño phenomenon to the atmosphere, has similar interannual quasi-period peaks to those of the Niño $3.4$ index. However, because the specific heat capacity of the atmosphere is small, the thermodynamic properties of the sea-level pressure field are affected not only by the underlying ocean but also by the high-frequency changes at the seasonal scale^39^. Therefore, the frequency spectrum of the SOI is significantly stronger than that of the Niño $3.4$ index. That is, the SOI data change more drastically than do the Niño $3.4$ index data, and this high-frequency random noise severely reduces the model’s ability to predict the SOI data. Although the EEMD method was used to decompose high-frequency components into low-frequency subcomponents, it still cannot reach the prediction level of the Niño $3.4$ index. However, the PCC value of the worst forecast still exceeded 0.5, which strongly proves the effectiveness of the EEMD-TCN model in SOI advance prediction.

## Evaluation and discussion

**Figure 6:**
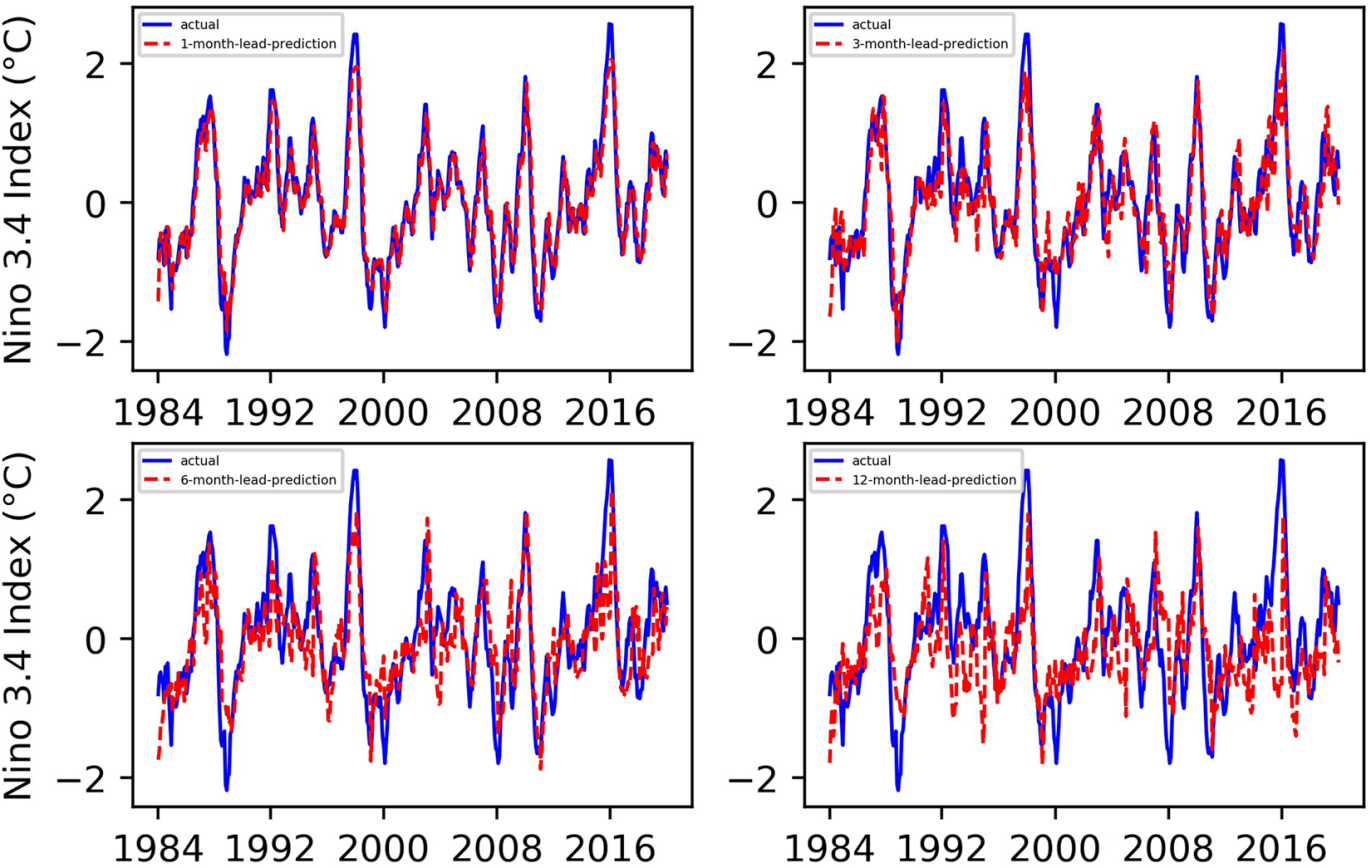
Predicted and actual values of the TCN-based Niño $3.4$ index for different month lead times.

**Figure 7:**
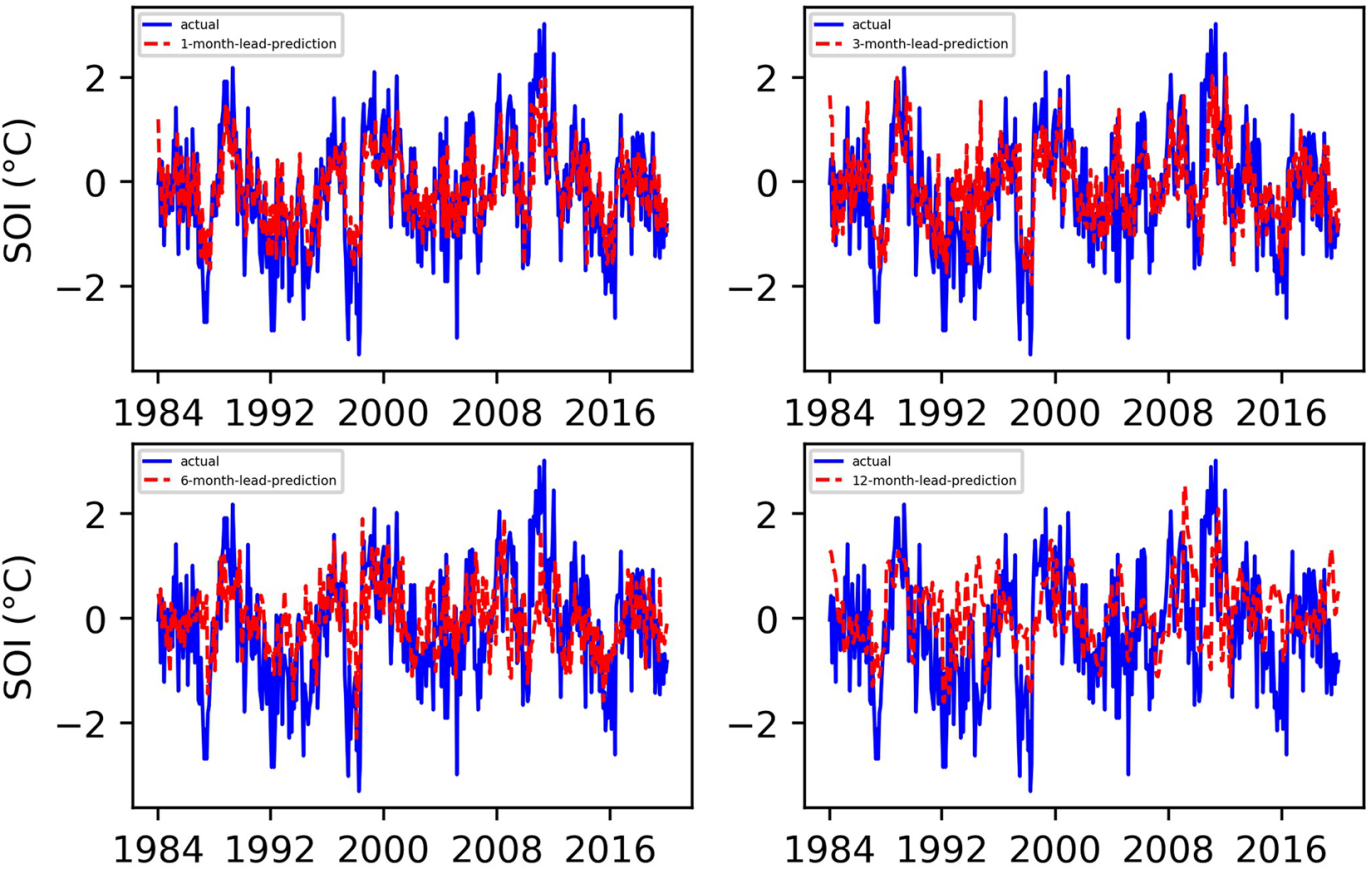
Predicted and actual values of the TCN-based SOI for different month lead times.

**Figure 8:**
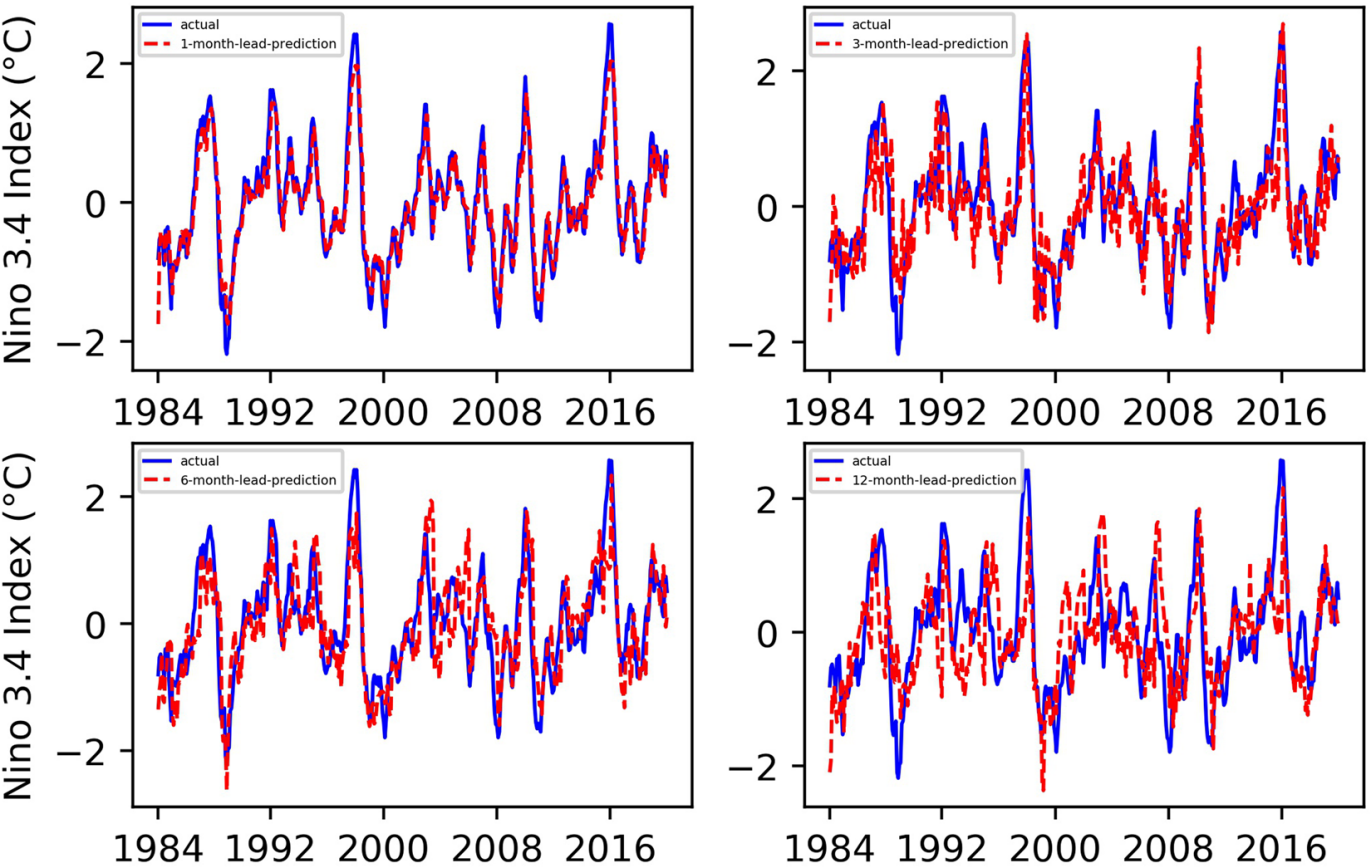
LSTM-based Niño $3.4$ index predicted and actual value curves.

**Figure 9:**
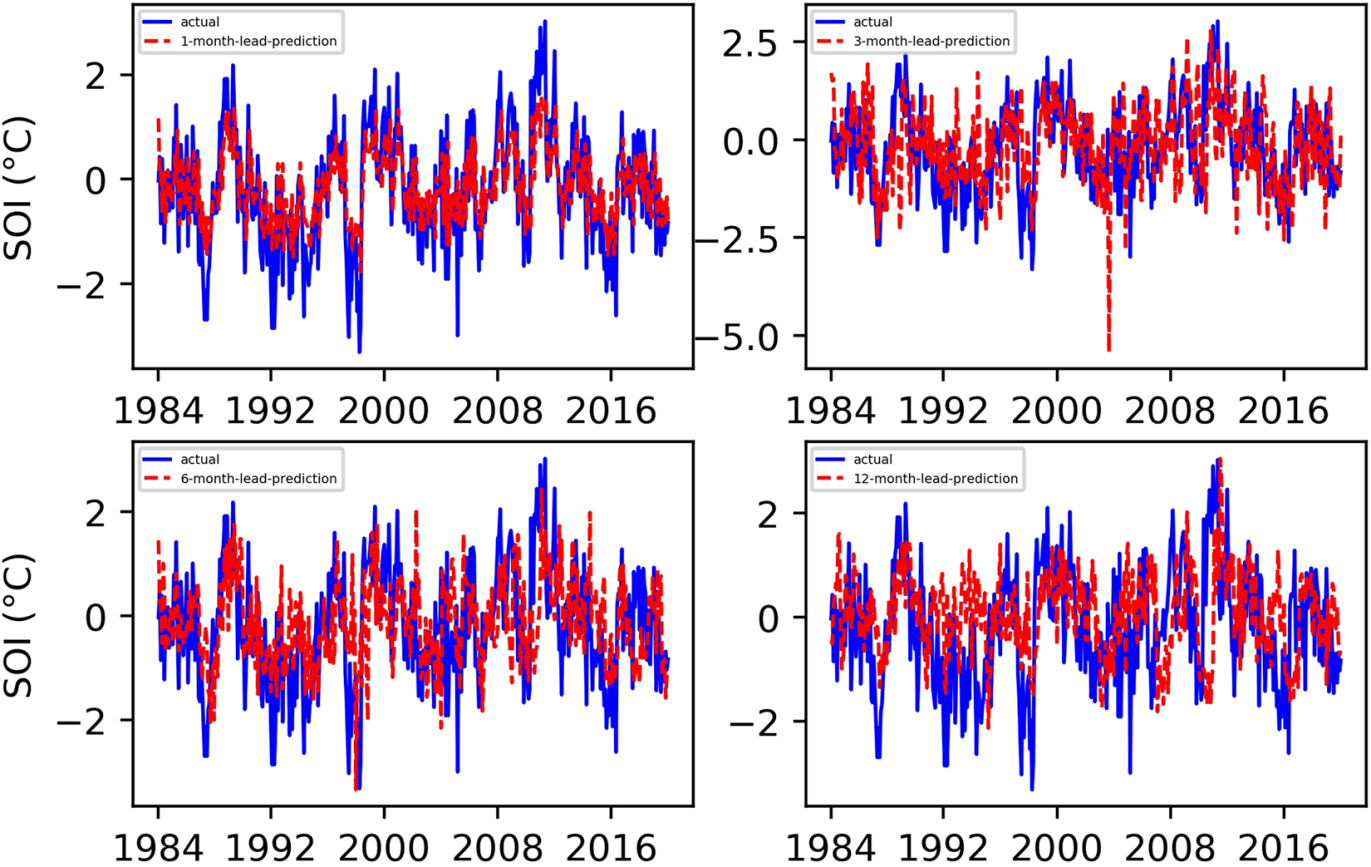
LSTM-based SOI predicted and actual value curves.

**Figure 10:**
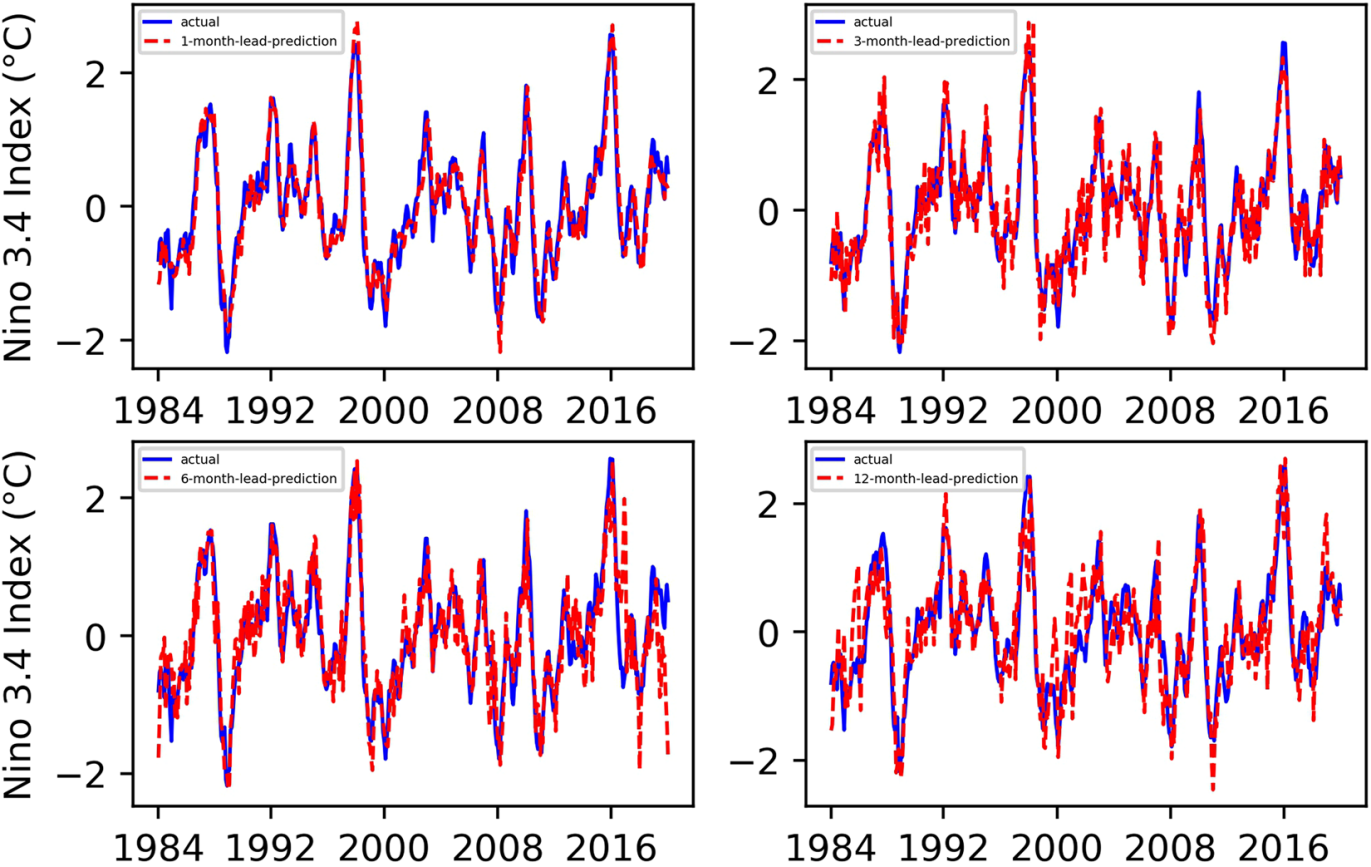
EEMD-LSTM-based Niño $3.4$ index predicted and actual value curves.

**Figure 11:**
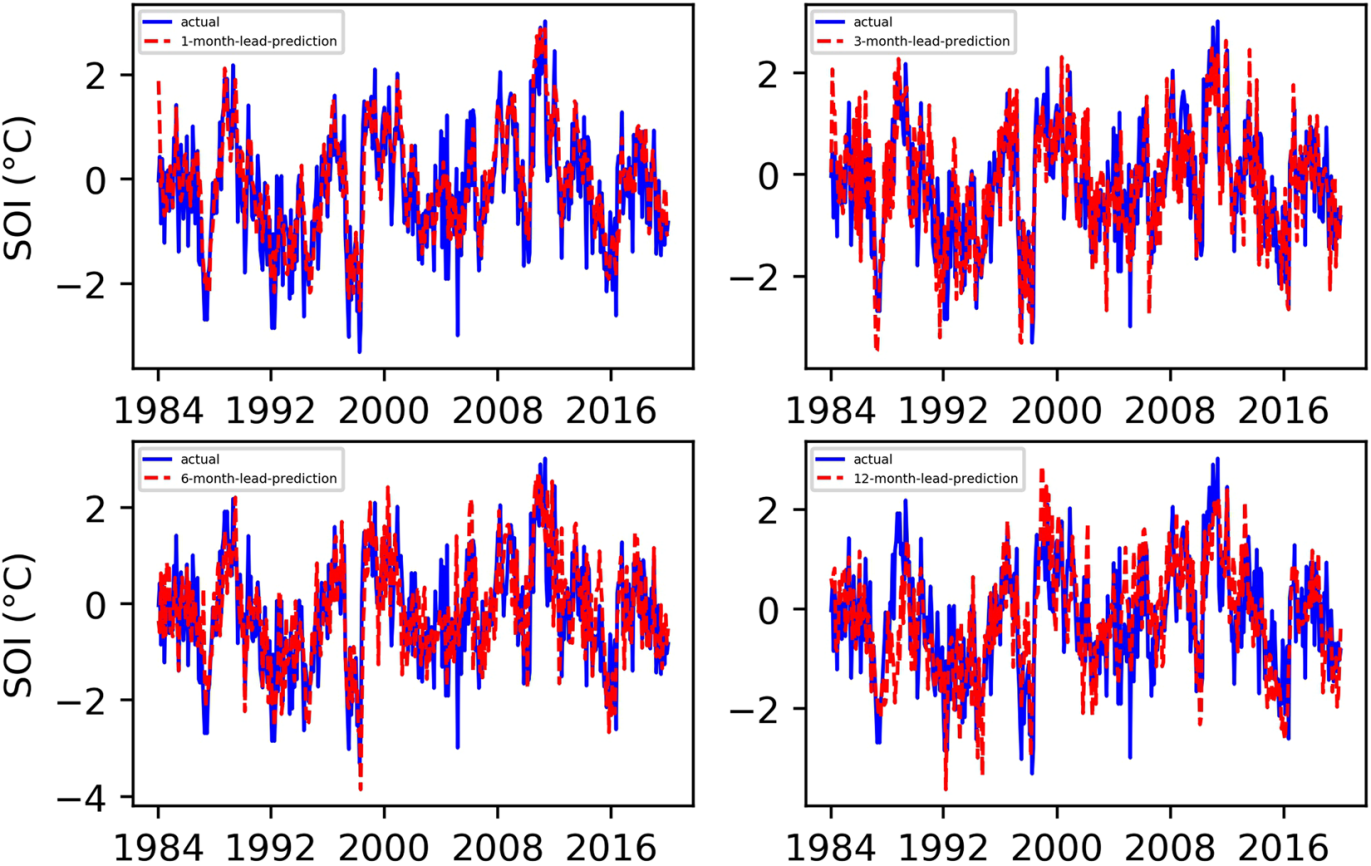
EEMD-LSTM-based SOI predicted and actual value curves.

**Figure 12:**
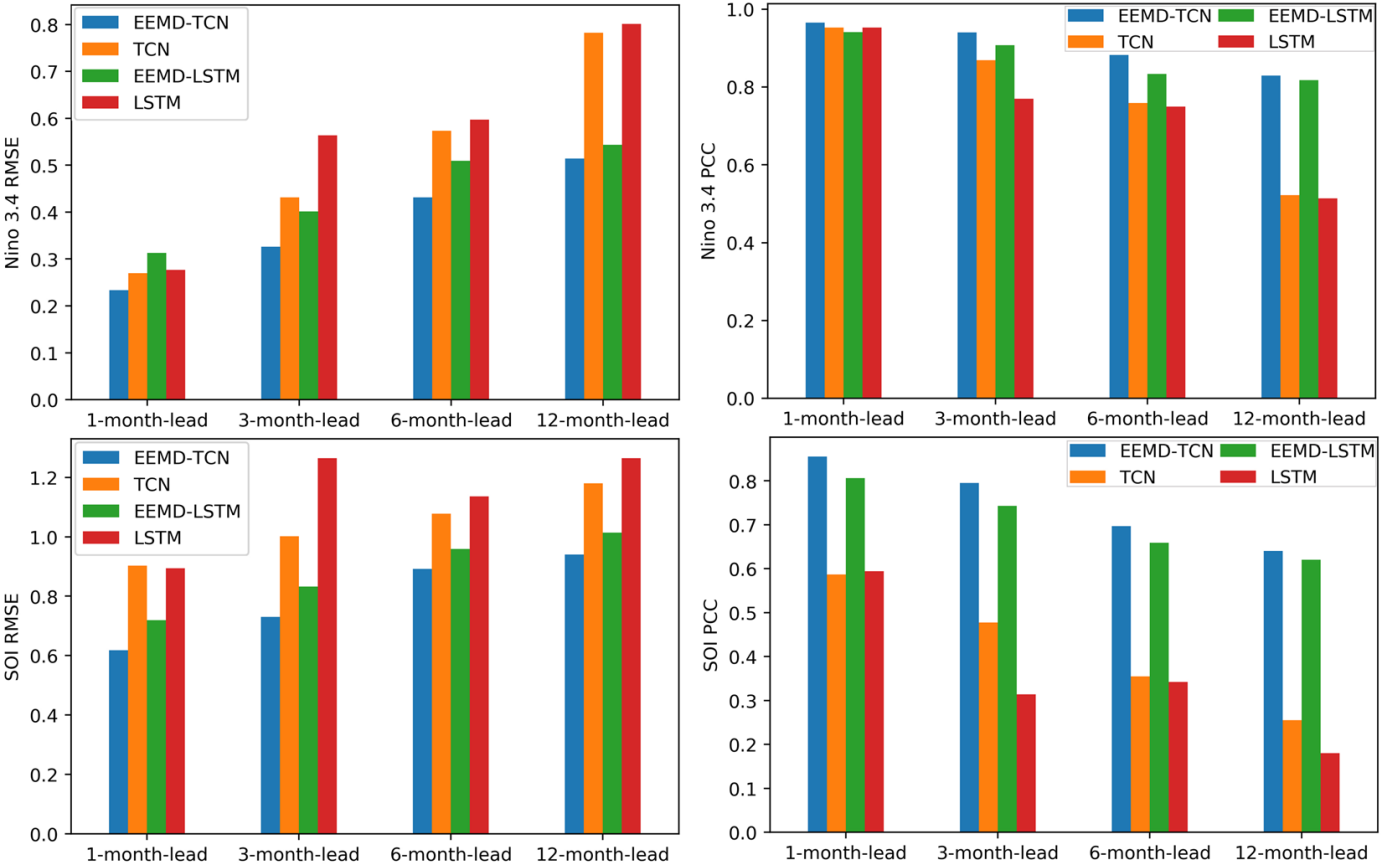
RMSE and PCC comparison of predicted and actual values of Niño $3.4$ index and SOI obtained using the TCN, EEMD-TCN, LSTM, and EEMD-LSTM methods.

To effectively evaluate the performance of our proposed “first EEMD and then TCN prediction” ENSO prediction approach, fully verifying the key role of EEMD decomposition in ENSO prediction, we carried out comparative experiments with the TCN, LSTM, and EEMD-LSTM. All comparative experimental data, as well as the training set and test set assignments, were the same as those of the EEMD-TCN-based ENSO prediction experiment. Figures 6 and 7 show the final Niño $3.4$ index and SOI results predicted by the classic TCN approach, Figs. 8 and 9 are the results predicted by the classical LSTM model, and Figs. 10 and 11 show the final Niño $3.4$ index and SOI results predicted by the EEMD-LSTM approach. For the TCN, LSTM and EEMD-LSTM comparative experiments, the number of iterations was still set to 7000, consistent with the number of EEMD-TCN iterations. In addition, the RMSE and PCC values of the predicted results of each comparative experiment were also calculated, as shown in Fig. 12.

From the aforementioned comparison experiments, the following can be concluded.

- As shown in Fig. 6, compared with the “first EEMD and then TCN prediction” method, the prediction result of the Niño $3.4$ index obtained by the pure TCN model is relatively poor. Especially at the points where there are strong El Niño or La Niña phenomena, there are large prediction errors, and the error becomes increasingly obvious as the advance prediction time increases.
- From Fig. 7, the SOI prediction results obtained by using the TCN were worse than those obtained by the EEMD-TCN algorithm, regardless whether considering a one-month-lead forecast or a 12-month-lead forecast. This result may be due to the high-frequency components contained in the SOI time series, which lower the prediction accuracy of the pure TCN model, further confirming our hypothesis that the EEMD-TCN technique can effectively improve the prediction accuracy achieved on high-frequency variation time series.
- As shown in Figs. 8 and 10, EEMD can also effectively improve the prediction accuracy of the LSTM model for the Niño $3.4$ index, especially at those times when there are strong El Niño or La Niña phenomena. However, compared with the TCN model, the overall performance of the LSTM model for the Niño $3.4$ index prediction is worse, which can also be verified from the RMSE and PCC values of the prediction results of the two (Fig. 12).
- On the basis of Figs. 9 and 11, for the overall SOI prediction accuracy, EEMD-LSTM is better than the LSTM model. However, as a whole, the SOI prediction accuracies obtained by the LSTM and EEMD-LSTM models are worse than the Niño $3.4$ index accuracies. This outcome is consistent with the prediction results of the EEMD-TCN and TCN models, which were determined on the basis of the high-frequency characteristics of the SOI data itself.
- In Fig. 12, the longer the advance forecasting time, the more obvious the advantages of EEMD, no matter whether the TCN model or LSTM model is used. Therefore, the conclusion is that decomposing a time series that mixes low- and high-frequency components into sub-components containing a single frequency and making separate predictions can effectively improve the accuracy of long-term advance prediction.
- As shown in Fig. 12, for the 1-, 3-, 6-, and 12-month-lead EEMD-TCN-based Niño $3.4$ index and SOI prediction, the RMSE values were the smallest and the PCC values were the highest compared with the corresponding results of the TCN, LSTM, and EEMD-LSTM methods. In addition, for the same test and validation data set and with the same number of training iterations, the LSTM model takes approximately 8 times longer than the TCN model on an RTX 2080Ti GPU. In other words, the EEMD-TCN was the best model for ENSO advance prediction in terms of the prediction accuracy and efficiency.

## Conclusions and Future Work

In view of the low accuracy of the current popular ENSO prediction methods, and considering Niño $3.4$ index and SOI reanalysis data containing many low- and high-frequency components, we proposed adopting a “first EEMD and then TCN prediction” hybrid approach, which decomposes the highly variable Niño $3.4$ index and SOI into relatively flat subcomponents and then uses the TCN model to predict each subcomponent in advance, finally combining the sub-prediction results to obtain the final ENSO prediction results. The Niño $3.4$ index and SOI reanalysis data from 1871 to 1973 were used for model training, and the data for 1984–2019 were predicted 1 month, 3 months, 6 months, and 12 months in advance. The results show that for both the Niño $3.4$ index and SOI reanalysis data, the accuracy of the 1-month-lead prediction was the highest, and the 12-month-lead prediction was the slowest. Specifically, for Niño $3.4$ index advance prediction, the curve obtained by the one-month-lead prediction had the highest degree of coincidence with the actual value, the RMSE was 0.2337, and the corresponding PCC was 0.9658; the curve predicted by the 12-month-lead prediction matched the actual curve the least, the RMSE was 0.5142, and the corresponding PCC was 0.8297; and for the SOI advance prediction, the RMSE of the one-month-lead prediction was 0.6180, and its corresponding PCC was 0.8556, while the accuracy of the 12-month-lead prediction was the worst, with an RMSE value of 0.9403 and a PCC value of 0.6406. Furthermore, the overall prediction accuracy on the Niño $3.4$ index was better than that on the SOI, which may have occurred because the SOI contains too many high-frequency components, causing the prediction to be difficult. The results of comparative experiments with the TCN, LSTM, and EEMD-LSTM methods showed that the EEMD-TCN provided the best overall prediction of both the Niño $3.4$ index and SOI in 1-, 3-, 6-, and 12-month-lead predictions among all the methods considered. In particular, the TCN not only had higher prediction accuracy for the time-series data but also had a simpler network structure and higher operating efficiency than those of the popular LSTM network.

However, at those times when there are strong El Niño or La Niña phenomena, the prediction errors of both the Niño $3.4$ index and SOI were relatively large, and the errors became increasingly obvious as the advance prediction time increased. In addition, the proposed EEMD-TCN approach could not overcome the “spring forecast obstacles” in dynamic forecasting, and the correlation coefficients between the 3- and 6-month-lead predicted results and actual values were significantly reduced. Recently, several studies^40–42^ used the physical-empirical model and/or statistical-dynamic model to improve the prediction of climate signals such as ENSO and Arctic Oscillation. The methods used in these studies consider both physical mechanism and numerical simulation, providing a new idea to improve the prediction accuracy of our EEMD-TCN model. Therefore, attempts to improve the prediction accuracy at time points with strong El Niño or La Niña phenomena, as well as overcoming the “spring forecast obstacles”, will be carried out in future research.

## Methods

### TCN

For the analysis of time-series data, the most commonly used neural network is the RNN^33^. RNN can employ the internal memories to process input time series, which is different from the traditional back-propagation (BP) neural network. However, the RNN model is generally not directly used for long-term memory calculation; thus, the improved RNN model known as LSTM was proposed^43^. LSTM can process sequences with thousands or even millions of time points, and has good processing ability even for long time series containing many high- and low-frequency components^44^. However, the latest research shows that the TCN, one of the members of the convolutional neural network (CNN)^45^ family, shows better performance than LSTM in processing very long sequences of inputs^46^.

The typical characteristics of TCN includes: (1) It can take a sequence of any length and output it as a sequence of the same length with the input, just like using an RNN; and (2) the convolution is a causal convolution, which means that there is no information “leakage” from future to past. To reach the first goal, the TCN uses a one-dimensional, fully convolutional network (1D FCN) architecture^46^. That is, each hidden layer will be padded zero to maintain the same length with the input layer. To achieve the second point, the causal convolution, where an output at time $t$ is convolved only with elements from time $t$ and earlier in the previous layer, is adopted. In short, TCN is the sum of 1D FCN and causal convolutions.

#### FCN

**Figure 13:**
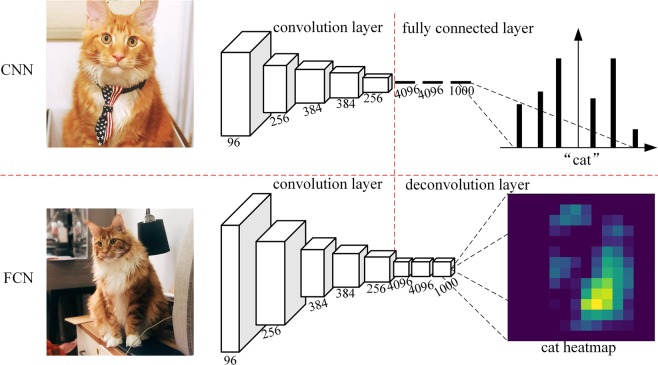
The difference between the CNN and FCN (the transforming of fully connected layers into convolutional layers by an FCN enables a classification net to output a heatmap).

Unlike the classic CNN, which uses a fully connected layer after the convolutional layer to obtain a fixed-length feature vector, the FCN uses the deconvolutional layers for the last convolutional layers^47^. That is, all the hidden layers in the neural network are convolutional layers, hence why it is named a “fully convolutional” network (Fig. 13).

The FCN can accept input images of any size, and its output has the same size as that of the input images thanks to the upsampling after the last convolutional layers, that is, deconvolution. Therefore, a prediction can be generated for each input pixel while preserving the spatial information in the original input image^46^. If the input images become 1D series data, then the FCN becomes a 1D FCN. Because the input and output of the FCN have the same size, the 1D FCN can produce an output with the same length as that of the input.

#### Causal convolutions

For sequence modelling, the main purpose is to predict some corresponding outputs ${{\rm{y}}}_{0},\ldots ,{y}_{T}$ at each time according to an input sequence ${{\rm{x}}}_{0},\ldots ,{x}_{t}$. If ${{\rm{y}}}_{t}$ depends only on ${{\rm{x}}}_{0},\ldots ,{x}_{t}$ and not on any future inputs ${{\rm{x}}}_{{\rm{t}}+1},\ldots ,{x}_{{\rm{T}}}$, then the goal is to find a network $F$ that minimizes the difference between the prediction and the actual outputs. That is, $min\{L[({y}_{0},\ldots ,{y}_{T}),f({x}_{0},\ldots ,{x}_{T})]\}$, where $L$ represents the loss between the actual outputs and predictions.

**Figure 14:**
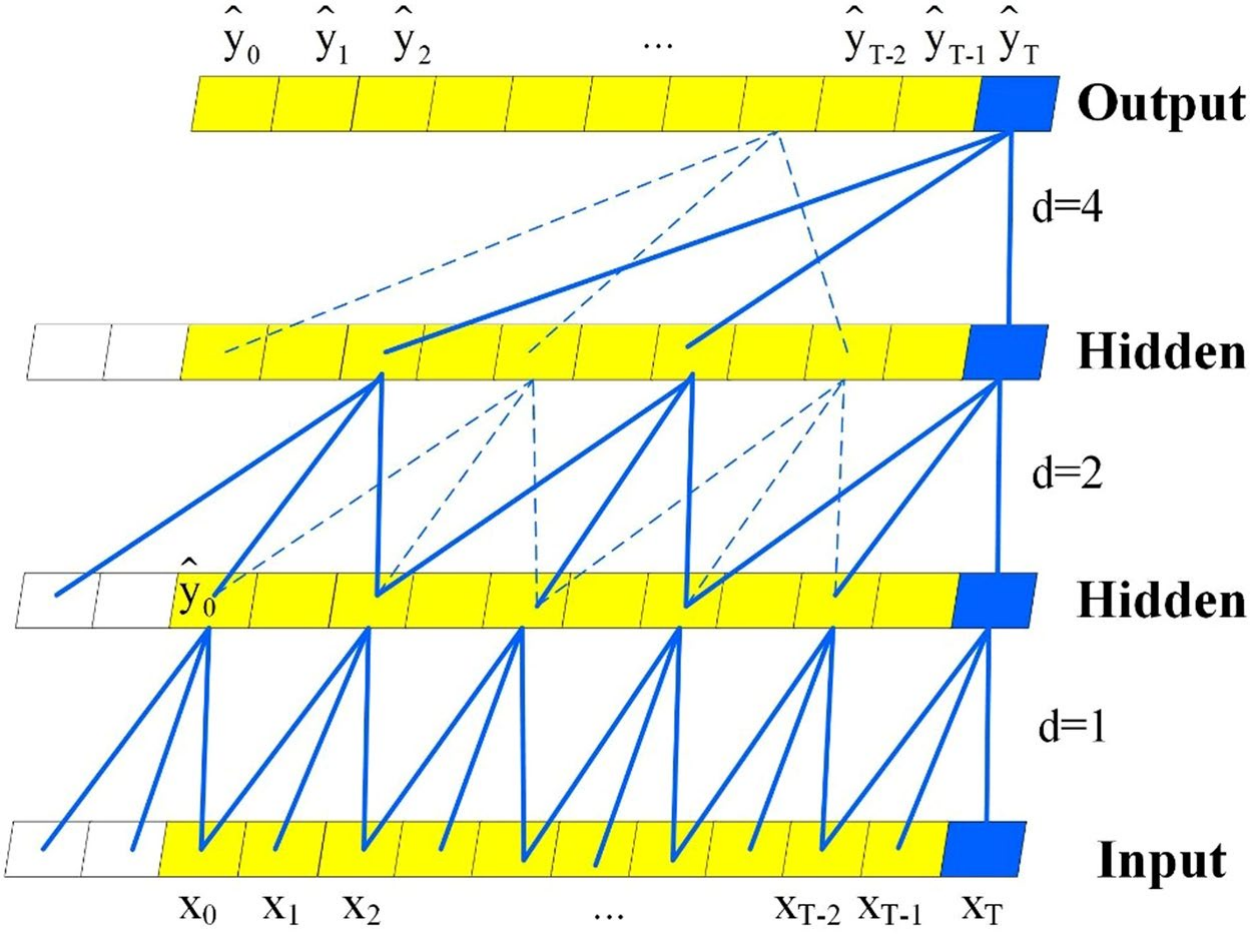
A dilated causal convolution with dilation factors d = 1, 2, 4 and a filter size k = 3.

The ordinary CNN is not suitable for addressing sequence problems because the input image size of a CNN must be fixed^48,49^; thus, a causal convolution was used. However, it is very challenging to directly apply a simple causal convolution to deal with long time series problems, because it can only look back at a history with a linear size in the depth of the network. To eliminate this problem, the dilated convolution, which enables an exponentially large receptive field^50^, is employed. The same points between the simple causal convolution and the dilated convolution are that both of them have the same size of the convolutional kernel and the same number of parameters, and the difference is that the dilated convolution has a dilation rate parameter to indicate the size of the dilation^51^. More formally, for a 1D sequence input ${\rm{X}}\,\in \,{R}^{n}$ and a filter ${\rm{f}}:\,\mathrm{\{0,}\ldots ,k-\mathrm{1\}}\to R$, the dilated convolution operation $F$ on elements $s$ of the sequence is defined as follows.

3 $${\rm{F}}({\rm{s}})=({\rm{X}}{\ast }_{{\rm{d}}}{\rm{f}})({\rm{s}})=\mathop{\sum }\limits_{i=0}^{k-1}f(i)\cdot {X}_{s-d\cdot i}$$

where $d$ denotes the dilation factor, $d$ is the filter size, and ${\rm{s}}\,-\,d\cdot i$ accounts for the direction of the past. Figure 14 illustrates the architectural elements in a TCN.

As shown in Fig. 14, when *d* = 1, the dilated convolution becomes a simple convolution; if we choose larger filter sizes *k* and increase the dilation factor *d*, the receptive field of the TCN can be increased. Therefore, we can use these methods to address long-sequence problems.

#### Residual connections

In addition, as the length of the time series increases, the TCN receptive field widens, resulting in the number of network layers and the number of filters per layer increasing, as the TCN receptive field depends on the network depth *n*, filter size *k*, and dilation factor *d*.

**Figure 15:**
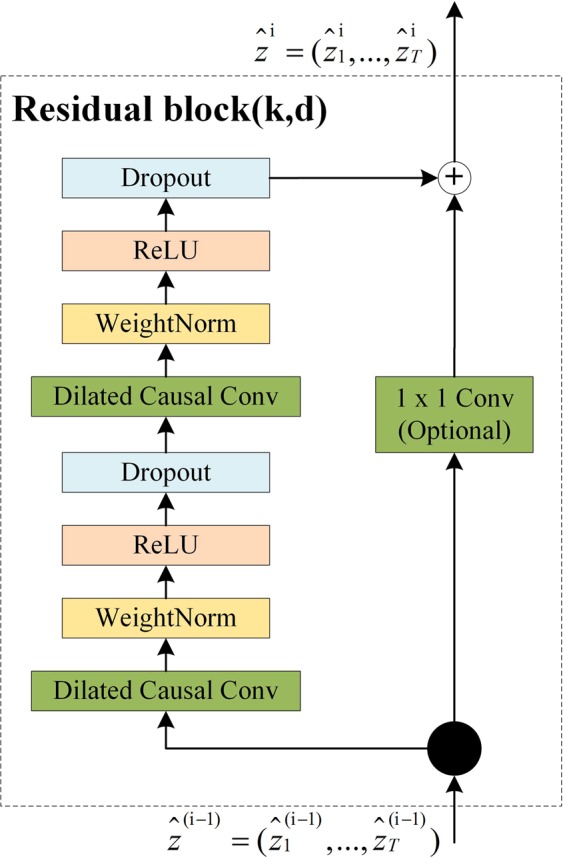
TCN residual block.

However, the major issue for very deep networks is exploding and/or vanishing gradients; the TCN model uses a generic residual module instead of a convolutional layer to avoid these problems. Figure 15 shows the residual block for a TCN.

In Fig. 15, the TCN model has two layers, i.e., a dilated causal convolution and non-linearity (ReLU), as well as weight normalization in between. In addition, a spatial dropout was added after each dilated convolution for regularization, and an additional $1\times 1$ convolution was adopted to ensure that the element-wise addition $\oplus$ received tensors of the same shape to resolve the difference in input and output widths.

### EEMD

**Figure 16:**
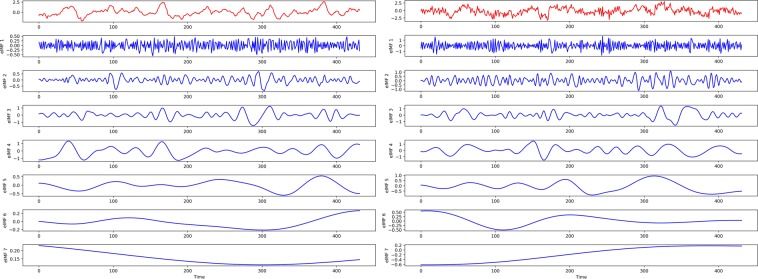
The original time series (red) and its IMFs (blue). The picture on the left is the Niño $3.4$ index, and the picture on the right is the SOI.

EEMD is an improved version of EMD that effectively overcomes the drawbacks of mode mixing in conventional EMD. Its principle is to add the normal distribution of white noise to the original signal subjected to EMD decomposition, then to use the spectral characteristics of the white noise uniform distribution to offset the specific spectrum loss of the original signal, and finally to eliminate the modal aliasing inherent in EMD^35^. After EEMD, the original high-volatility time series can be divided into some adaptive orthogonal components, called IMFs, which cannot maintain the original characteristics but greatly reduce the annualized volatility. Figure 16 shows the original Niño $3.4$ index and SOI series, as well as their EEMD-decomposed components. It can be seen that after EEMD, each IMF component of the Niño $3.4$ index and SOI series contains only one frequency component, which can effectively improve the prediction accuracy of the model.

### EEMD-TCN-based ENSO index series prediction

After the original Niño $3.4$ index and SOI series were decomposed into multiple IMFs components, each single component could be predicted using the TCN model and combined to obtain the final prediction result. For the TCN-based single component prediction, the core problem is to determine the network parameters, including the dilation factor $d$, the filter size $k$, and the minimum network depth $n$^46^, based on data characteristics to obtain accurate time-series prediction results.

#### Dilation factor

The dilation factor $d$ generally increases as the depth $n$ of the network increases. Their relationship can be expressed by the following formula^52^:

4 $${d}_{i}={2}^{i}\mathrm{,1}\le i\le n$$

where $i$ represents the *i*-th layer and $n$ represents the total number of dilated causal convolutional layers.

#### Filter size

The filter size, also known as the convolutional kernel size, varies with the dilation factor $d$, and their relationship can be expressed by the following formula^53^:

5 $${k}_{i+1}={d}_{i}\ast ({k}_{i}-1)+\mathrm{1,}\,1\le i\le n,{k}_{1}\in {N}^{\ast }$$

where $i$ represents the $i$-th layer and $n$ represents the total number of dilated causal convolutional layers. In general, the initial size of the filter is ${k}_{1}=2$ by default, but it can be set to other values depending on the data situation.

#### Minimum network depth

In the TCN model, the minimum depth of the TCN directly affects the receptive field, which is determined by the following formula:

6 $$receptiveField=nb\_stacks\_of\_residual\_block\ast {k}_{1}\ast {d}_{n}$$

where $nb\_stacks\_of\_residual\_block$ is the number of stacks of residual blocks to use, which is set to 1 by default, ${k}_{1}$ is the initial size of the filter, and ${d}_{n}$ is the dilation factor of the *n*-th dilated causal convolutional layer; *n* represents the total number of dilated causal convolutional layers. Therefore, it is necessary to comprehensively consider the length of the input sequence and the size of the receptive field to obtain a reasonable minimum network depth.
